# Letter in Response to ‘Standardised Request and Contrast Consent Forms to Enhance Clinical Learning in Radiography Education’

**DOI:** 10.1002/jmrs.70077

**Published:** 2026-03-16

**Authors:** Clare L. Singh, Kelly Bentley‐Spuur, Lorraine Rose, Pauletta Irwin, James Yun, Minh Chau

**Affiliations:** ^1^ Charles Sturt University Wagga Wagga New South Wales Australia; ^2^ Faculty of Medicine and Health The University of New South Wales Sydney Australia; ^3^ Department of Immunology Prince of Wales Hospital Sydney Australia; ^4^ South Eastern Sydney Immunology Laboratory NSW Health Pathology Sydney Australia

## Abstract

This letter to the Editor is in response to the paper ‘Standardise Request and Contrast Consent Forms to Enhance Clinical Learning in Radiography Education’ by Nocum et al. The writers applaud the authors for addressing the importance of documentation in learning and teaching with this practical and student‐centred approach. We offer a recommendation for consideration regarding terminology to ensure that the form content is aligned with international consensus on hypersensitivity. This minor adjustment would, in our view, strengthen what is an invaluable tool in radiography education.

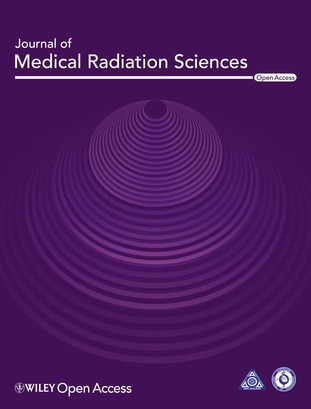

We read with interest the article by Nocum et al. regarding the development and implementation of standardised request and consent forms in the educational setting [1]. We commend the authors for this much needed contribution. Embedding authentic documentation into teaching and simulation aligns well with constructivist learning principles and proactively strengthens gaps in clinical learning [2].

The authors rightly identify that standardised forms act as a powerful pedagogical tool. In the context of education and standardisation, we suggest minor but significant refinement to the terminology used, specifically in the contrast form. The item ‘Have you had an allergic reaction to x‐ray contrast media?’ could be rephrased to reflect more clinically current terminology [3, 4]. It is acknowledged that ‘hypersensitivity’ is not a patient‐centric term; however, moving away from the ‘contrast allergy’ label (unless confirmed) is important in preventing long‐term negative impact on patient care [5, 6].

From an educational perspective, this distinction is imperative. As the authors are focused on enhancing clinical learning, they are in a unique position to help dismantle the self‐perpetuating cycle of the ‘contrast allergy’ label [7]. Radiographers are frequently involved in eliciting patient histories and initiating documentation. Education research in CT training shows that structured, authentic learning tools and guided prompts can meaningfully shape students' preparedness for documentation and clinical decision‐making [8]. The tool developed provides an opportunity to move away from ‘contrast allergy’ terminology, supporting students in documenting reactions and providing patients with accurate information. Our recent socio‐ecological scoping review on contrast hypersensitivity documentation highlights how inconsistent terminology contributes to the persistence of inaccurate records (‘contrast allergy’ flag) [7]. Inadvertently, this small change in the form can work towards preventing known issues such as contrast avoidance, unnecessary pre‐medication, and increased healthcare costs [5, 9].

It was observed that the development of the form was informed by the Royal Australian and New Zealand College of Radiologists (RANZCR) guidelines. As these guidelines are due for review, the European Society of Urogenital Radiology and Canadian Association of Radiologists in collaboration with the Canadian Society of Allergy and Clinical Immunology provide a more contemporary approach with respect to reaction classifications and documentation expectations [10, 11].

Students must understand the nuances of patient histories: whether a symptom resembles an allergy, whether it occurred during the first or subsequent administrations of contrast, whether the contrast was iodinated or gadolinium‐based, and whether the reaction was allergologically confirmed [12, 13]. All of this information affects decision‐making with respect to contrast. Students (and practitioners) also need to understand the importance of side chains in contrast and how this can affect cross‐reactivity [14, 15].

Nocum et al. have provided an excellent foundation for improving the learning and teaching of clinical documentation. In doing so, they have also revealed a major issue with current contrast consent forms. We thank the authors for their leadership in this space. Refining the terminology will align with current up‐to‐date and evidence‐based international guidelines and consensus and ultimately benefit patient safety.

## Conflicts of Interest

The authors declare no conflicts of interest.

## Data Availability

The authors have nothing to report.
